# To what extent do supervised drug consumption services incorporate non-injection routes of administration? A systematic scoping review documenting existing facilities

**DOI:** 10.1186/s12954-020-00414-y

**Published:** 2020-10-07

**Authors:** Kelsey A. Speed, Nicole D. Gehring, Katherine Launier, Daniel O’Brien, Sandy Campbell, Elaine Hyshka

**Affiliations:** 1 School of Public Health, University of Alberta, Edmonton, AB Canada; 2 Inner City Health and Wellness Program, Royal Alexandra Hospital, Edmonton, AB Canada; 3 John W. Scott Health Sciences Library, University of Alberta, Edmonton, AB Canada

**Keywords:** Supervised consumption service, Drug consumption room, Drug policy, Harm reduction, Safer inhalation

## Abstract

**Background:**

Most of the existing research on supervised consumption services (SCS) is focused on injection drug use. Less is known about the applicability of SCS for people who consume drugs orally, intranasally, or through inhalation. This is problematic because people who use drugs through modes other than injection are also at risk of overdose death and other harm, and experience barriers accessing health and social services. We aimed to describe existing SCS models that accommodate these alternate routes of drug consumption, and synthesize available information on characteristics of program participants.

**Methods:**

We conducted a systematic scoping review of 9 peer-reviewed and 13 grey literature databases on SCS that incorporate non-injection routes of consumption. We screened 22,882 titles, and excluded 22,843 (99.8%) articles. We ultimately included 39 (0.2%) full-text articles; 28 (72%) of these articles explicitly identified SCS that permit alternate routes of consumption and 21 (54%) discussed characteristics of participants who consume drugs through non-injection routes. Data on study characteristics, terms and definitions, and site and program participant characteristics were extracted and double-coded. Extracted data were analyzed using descriptive statistics and narrative synthesis.

**Results:**

Included articles describe 48 SCS that permit non-injection routes of consumption, most of which were located in Germany. The majority of these SCS were legally sanctioned and had models of care that were largely comparable to supervised injection services. Notable differences included physical infrastructure such as ventilated rooms or outdoor areas to accommodate inhalation, and shorter time limits on non-injection drug consumption episodes. Program participants engaging in non-injection forms of consumption were typically men over the age of 30 and structurally vulnerable (e.g., experiencing homelessness or unstable housing).

**Conclusions:**

Extant academic and grey literature indicates that site characteristics and demographics of program participants of SCS that permit non-injection routes of consumption largely reflect those of supervised injection services. Further research on the range of existing SCS that incorporate non-injection routes of consumption is needed to ensure high quality service provision, and improved health outcomes for people who consume drugs via oral, intranasal, and inhalation routes.

## Introduction

Supervised consumption services (SCS) “…are protected places used for the hygienic consumption of pre-obtained drugs in a non-judgemental environment and under the supervision of trained staff” [1] (p. 2). As of December 2018, there were 117 SCS operating globally [2]. Studies of SCS demonstrate that monitoring injection drug consumption reduces overdose risk and other negative health outcomes, helps connect people to health and social services, and can contribute to reductions in improperly discarded syringes and other public disorder [3, 4]. However, the majority of scientific literature on SCS is derived from two facilities: Insite in Vancouver, Canada and the Medically Supervised Injecting Centre in Sydney, Australia which are targeted specifically for people who inject drugs [3, 4]. Much less is known about the practice of supervising non-injection forms of consumption and SCS models that accommodate people taking drugs by oral, intranasal, and inhaled routes. This is problematic because people who inject drugs are only a subsection of the overall population of people who use illegal drugs. Globally, out of approximately 100 million people who consumed opioids, amphetamines, and cocaine in 2016/2017, only 11 million consumed their drugs through injection [5].

Injection is typically the riskiest route of illegal drug administration, and people who inject drugs have an amplified risk of human immunodeficiency virus (HIV), hepatitis C, and overdose compared to those who consume drugs via non-injection routes [6]. Nevertheless, many people who take drugs by non-injection routes of consumption still experience harm. Fatal overdoses associated with illegal drug consumption by inhalation, intranasal, and oral routes have been documented in multiple settings [6–8]. In Canada and the USA, widespread contamination of the illegal drug market with clandestinely-produced synthetic opioids [9] has increased the risk of overdose for people who consume drugs via non-injection routes [10, 11]. Beyond overdose, evidence suggests that, when shared, pipes used for drug inhalation are a potential vector for hepatitis C transmission [12, 13], as is shared equipment for intranasal consumption (e.g., straws) [13]. Smoking drugs may also increase a person’s risk of HIV seroconversion [14], though the specific mechanisms for this association remain unclear. People engaged in public non-injection drug consumption report experiencing violence when using in public areas, from police and others [15, 16]. SCS that incorporate non-injection routes of consumption have the potential to reduce the risk of these harms through provision of emergency medical care, a safe environment, and sterile smoking and intranasal consumption supplies [17–20]. Additionally, providing access to SCS that incorporate non-injection routes of consumption may reduce the risk of transitioning to injection [21]. Prior research suggests that contact with health services can help reduce transitions from intranasal consumption to injection [22]. Harm reduction education from trained SCS staff could also help support people who inject drugs in transitioning to less risky modes of consumption. Excluding non-injection routes of drug consumption from SCS may undermine harm reduction efforts and expose people who use illegal drugs to preventable harms.

While the characteristics of people who consume drugs through non-injection routes of consumption have been described [23–26], less is known about the subpopulation of people who access SCS for oral, intranasal, or inhaled drug use. Recent evidence from a supervised consumption service in Canada demonstrated that program participants were predominantly Indigenous, male, and between the ages of 20–39 [17]. Whether these demographic characteristics are consistent in other SCS that offer non-injection routes of consumption is currently unknown. Many SCS in Europe permit non-injection routes of drug consumption [27] and SCS in North America are increasingly incorporating alternate routes of consumption. However, a comprehensive understanding of the characteristics of these SCS is lacking, potentially hindering the implementation of SCS for individuals who use non-injection routes of consumption. To address this gap, we conducted a systematic scoping review of the peer-reviewed and grey literature to describe the extent to which SCS monitor non-injection routes of drug consumption. Our specific objectives were to (1) identify SCS that incorporate non-injection routes of illegal drug consumption; (2) describe the service models and the characteristics of people who use SCS for inhalation, oral, or intranasal substance use; and (3) outline existing knowledge gaps on supervising non-injection routes of consumption to facilitate implementation and evaluation of new SCS models that meet the needs of all people who use illegal drugs.

## Methods

Our study design was adapted from the scoping review framework outlined by Arksey and O’Malley [28], and we report our results using the PRISMA Extension for Scoping Reviews (PRISMA-ScR) checklist [29]. No formal study protocol was published prior to conducting this study.

### Search strategy

There is no standard nomenclature for describing SCS in the literature. Accordingly, we developed a broad search strategy to capture all possible English terms used to describe these services in peer-reviewed and grey literature. Team members with detailed knowledge of harm reduction research developed the search terms, which were verified by an external expert with clinical and scientific expertise in the area. We conducted a pilot search, which helped us refine our search terms. SC further refined these terms, and executed a search on the peer-reviewed databases outlined in Table 1 using controlled vocabulary (e.g., MeSH, Emtree, etc.) and key words representing the concepts “supervised drug consumption” and “safer smoking” on September 12, 2017. No other limits were applied. The detailed search strategy applied to OVID Medline is available in Table 2 (see Additional file 1 for the remaining database search strategies). KS searched the first 10 pages of the grey literature resources outlined in Table 1 for any relevant articles available in English between October 4 and December 22, 2017. A list of key terms adapted from the peer-reviewed search strategy is provided in Additional file 2.

**Table 1:** Resources searched in the scoping review

Peer-reviewed database	Grey literature
OVID Medline (1946–current)	Google
OVID EMBASE (1974–current)	Google Scholar
OVID PsycINFO (1806–current)	International Network of Drug Consumption Rooms
EBSCO CINAHL	European Monitoring Centre for Drugs and Drug Addiction
PROSPERO	Archiv-It
Proquest Dissertations and Theses Global	Health Systems Evidence
Web of Science (SciEXPANDED)	Leading Practices Database
Cochrane Central Register of Controlled Trials	Harm Reduction International
Cochrane Database of Systematic Reviews	Bielefeld Academic Search Engine
	Grey Literature Report
	WorldCat
	AMICUS
	British Library
	Grey Literature Report

**Table 2:** Search strategy used for OVID Medline

#	Search statement	Results
1	((supervised or safe or safer) adj (injection or injecting or inhalation or inhaling or smoking or snorting or intranasal)).mp.	787
2	"drug consumption room"/ or ((injection or injecting or inhalation or inhaling or consumption or smoking or snorting or intranasal) adj (room or rooms or facility or facilities or service* or center or centers or centre or centres)).mp.	816
3	(((safe* or supervised) adj1 consumption) and (drug or drugs or opioid* or addict* or harm reduction or overdose*)).mp.	150
4	"fixing room*".mp.	1
5	"overdose prevention site*".mp.	19
6	1 or 2 or 3 or 4 or 5	1430
7	(airport or airports or operating room* or alcohol consump*).mp. or exp Operating Rooms/or exp Alcohol Drinking/ or exp Anaesthesia/or exp Ophthalmology/or *"Needlestick Injuries"/or *Smoking/or *Vaccines/	217,614
8	(ecigarette* or e-cigarette or cigarette smoking or (smoking adj2 cessation) or secondhand smok* or second handsmok* or "stop smoking" or vaccin* or immuniz* or operat* room* or surgical).mp.	1,914,462
9	7 or 8	2,043,582
10	6 not 9	1006
11	remove duplicates from 10	990
12	limit 11 to animals	61
13	limit 12 to humans	19
14	12 not 13	42
15	11 not 14	948
16	limit 15 to dt = 20170101–20170912	49
17	15	948
18	limit 17 to yr = "1860–2016"	642
19	16 or 18	690

We assessed the final search results against a list of exemplar articles provided in Additional file 3 to verify the scope of the search strategy. All of the articles except one commentary [21] were identified through the search of peer-reviewed databases. Collins et al. (2005) [21] was identified during a hand search of the reference lists of included articles, despite ultimately being excluded from the review based on the article type (an exclusion criterion; Table 3). Therefore, we believe our search strategy was sufficiently comprehensive to address our objectives.

**Table 3:** Inclusion and exclusion criteria for screening

Articles will be *included* if they:	Describe the rationale for incorporating non-injectable forms of drug consumption into supervised drug consumption service models; and/or Discuss the existence of supervised consumption services that allow program participants to consume drugs through forms other than injection; and/or Describe supervised consumption service models that allow program participants to consume drugs through forms other than injection (smoking, snorting, intranasal, inhalation, oral consumption, etc.); and/or Describe the real or potential impact of allowing non-injectable forms of drug consumption within supervised drug consumption services for program participants/potential participants. All study designs, reports, and book chapters that present or review research were eligible for inclusion.
Articles will be *excluded* if they:	Describe services or interventions related to the supervised consumption of legal drugs (e.g., tobacco, alcohol, medical cannabis, prescription drugs consumed as indicated) only; and/or Describe supervised consumption of drugs for the purposes of research on the physiological or behavioural effects of the drug; and/or Describe services or interventions related to the consumption of illegal drugs (safer inhalation kits, syringe distribution, take home naloxone, etc.) but do not provide a supervised setting for consumption; and/or Address supervised consumption of injection drugs only; and/or Describe supervised consumption of opioid agonist treatment only (including prescription diacetylmorphine, hydromorphone, oral morphine, buprenorphine/naloxone, methadone, etc.); and/or Are not available in English; and/or Are conference abstracts, commentaries, editorials, letters, or media articles; and/or Are not available publicly or through the University of Alberta holdings.

### Screening

We managed the articles from the peer-reviewed database search in RefWorks (*n* = 2619), and removed duplicates. We then transferred the remaining articles to Covidence, which is a Cochrane Collaboration recommended systematic review platform, where we removed more duplicates prior to screening (1213 total duplicates removed). KS and one of KL and NG independently screened the peer-reviewed literature by assessing the title and abstract of each article (*n* = 1552), followed by the full text article (*n* = 807), against the inclusion and exclusion criteria provided in Table 3.

KS identified potentially relevant articles during the grey literature search, and KS, DOB, and HB screened the title, abstract, and/or full-text (*n* = 4799) against the same inclusion and exclusion criteria as the peer-reviewed literature. As texts available through the grey literature search often did not provide an abstract, we did not conduct two-step screening as we did with the peer-reviewed literature. Each article was assessed by one of KS, DOB, or HB.

KS and AP cross-checked three external reference lists related to SCS for any potentially relevant articles that were not captured in the peer-reviewed databases and grey literature searches. These reference lists each consisted of a collection of articles related to SCS that were compiled by external organizations, and were not considered articles in-and-of themselves. KS, DOB, and NG hand searched the bibliographies of the included studies for any potentially relevant articles that were not captured previously.

This manuscript describes a subset of the articles that discuss SCS that permit non-injection routes of consumption. To establish this subset of articles, KS and NG screened the full-text articles by independently assessing them against the following inclusion criteria: (1) at least one SCS was identified by name as incorporating non-injection routes of consumption; and (2) information was provided about characteristics of program participants who use drugs orally, intranasally, or by inhalation by describing this subpopulation separately from those who inject drugs, or by clarifying that participant characteristics were the same across modes of drug consumption (without necessarily identifying the SCS by name as incorporating non-injection routes of consumption).

**Fig. 1:**
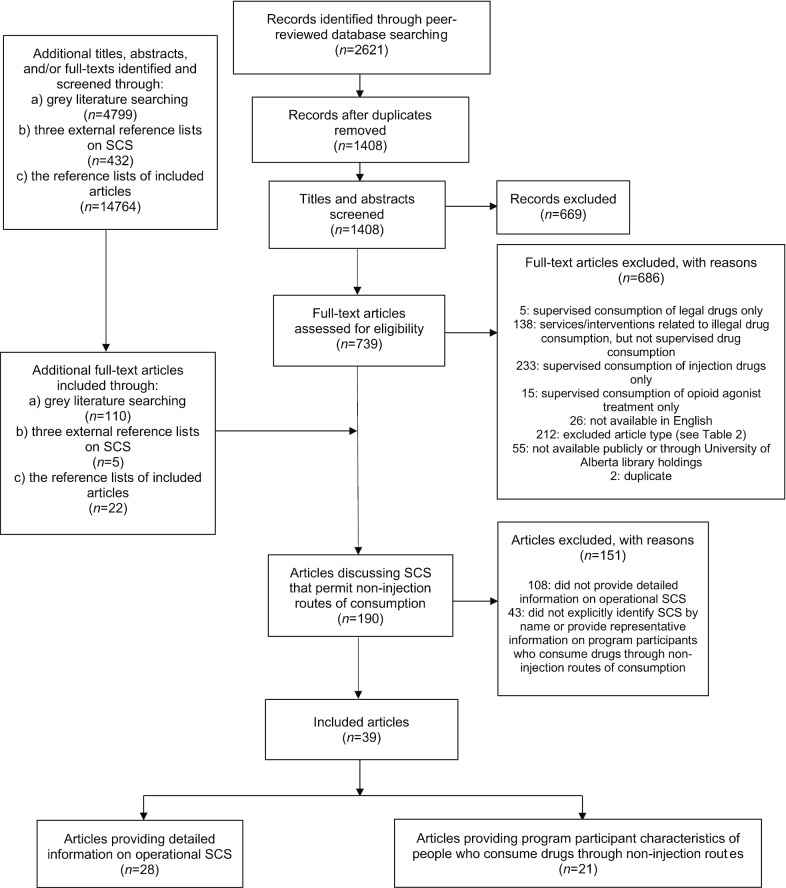
Screening flow chart

Any discrepancies in screening were discussed among the reviewers, and KS made the final decision in consultation with EH on any articles that did not clearly fit within either the inclusion or exclusion criteria. Reasons for exclusion and the final number of included articles are provided in Fig. 1.

### Data extraction

We developed a data extraction sheet based on the overall study objectives. KS and KL piloted the data extraction sheet by both extracting data from the same five articles. We refined the data extraction sheet in response to inconsistencies between coders. KS and KL then re-coded the same five articles and revised the variable definitions as required until we reached a consensus on the data extraction for these five articles. KS and KL then coded another 10 articles in order to finalize the data extraction sheet. The following data was extracted for each article: study characteristics (title, author(s), year of publication, home country of lead author, study design, and data type), SCS terms and definitions, SCS site characteristics (location, name, legal status, consumption route, model, layout, hours, staffing, rules and eligibility criteria, target population, number of visitors, services provided, and operational costs), and program participant characteristics (age, gender/sex, ethnicity, length of drug use, housing status). KS, KL, DOB, and NG double-coded the remaining included articles (two coders independently extracted data for each article). The coders discussed any disagreements in coding until a consensus was reached. If the two coders were unable to reach a consensus, a third team member was consulted and made the final decision.

### Data analysis

KS conducted the analysis of the extracted data using Microsoft Excel for Mac, Version 15.32, using descriptive statistics (counts and frequencies) to summarize patterns across studies, and narrative synthesis to summarize the extracted text. Narrative synthesis is particularly useful when the objectives, methods, and synthesis provided by the included articles are heterogeneous [30]. We adapted the narrative synthesis protocol for systematic reviews developed by Popay et al. (2006) by applying two of their identified steps, “developing a preliminary synthesis” and “exploring relationships in the data” (p.12). The other two steps, “developing a theoretical model of how the interventions work, why and for whom” and “assessing the robustness of the synthesis product” (p. 12), were not relevant to the objectives of the present study. NG verified the accuracy of the analysis.

### Included articles

Figure 1 details the literature screening process (further detail is provided in Additional file 4). Overall, our scoping review identified 193 articles that mentioned supervising non-injection routes of drug consumption in some capacity; we ultimately included 39 articles that met our objectives for this analysis. Of the included articles, 28 (72%) identified at least one SCS supervising non-injection routes of consumption, and 21 articles (54%) provided program participant characteristics.

Of the 39 included articles, over half were considered “grey” literature (*n* = 23; 59%), and the remaining articles were peer-reviewed literature (*n* = 16; 41%). Half of the included articles were non-empirical and provided neither qualitative nor quantitative data (*n* = 21; 54%), while the remaining articles provided qualitative data (*n* = 6; 15%), quantitative data (*n* = 9; 23%), or both qualitative and quantitative data (*n* = 3; 8%). Additional file 5 details the included articles on identified SCS that incorporate alternative routes of consumption, and Additional file 6 provides information on the included articles providing representative data on program participant characteristics.

## Results

There were many terms used in the included articles to discuss formal spaces where people can consume illegal drugs via non-injection routes under the supervision of trained staff, and these terms were often used interchangeably in the literature. Overall, 65 unique terms were used throughout the 39 articles included in this study. “Drug consumption room” was the term used the most frequently (*n* = 22), followed by “supervised injection facility” (*n* = 10), “consumption room” (*n* = 10), and “drug consumption facility” (*n* = 9). Other terms, such as “supervised inhalation facility,” “drug assistance service,” and “overdose prevention site” were also used.

### Characteristics of existing SCS

**Fig. 2:**
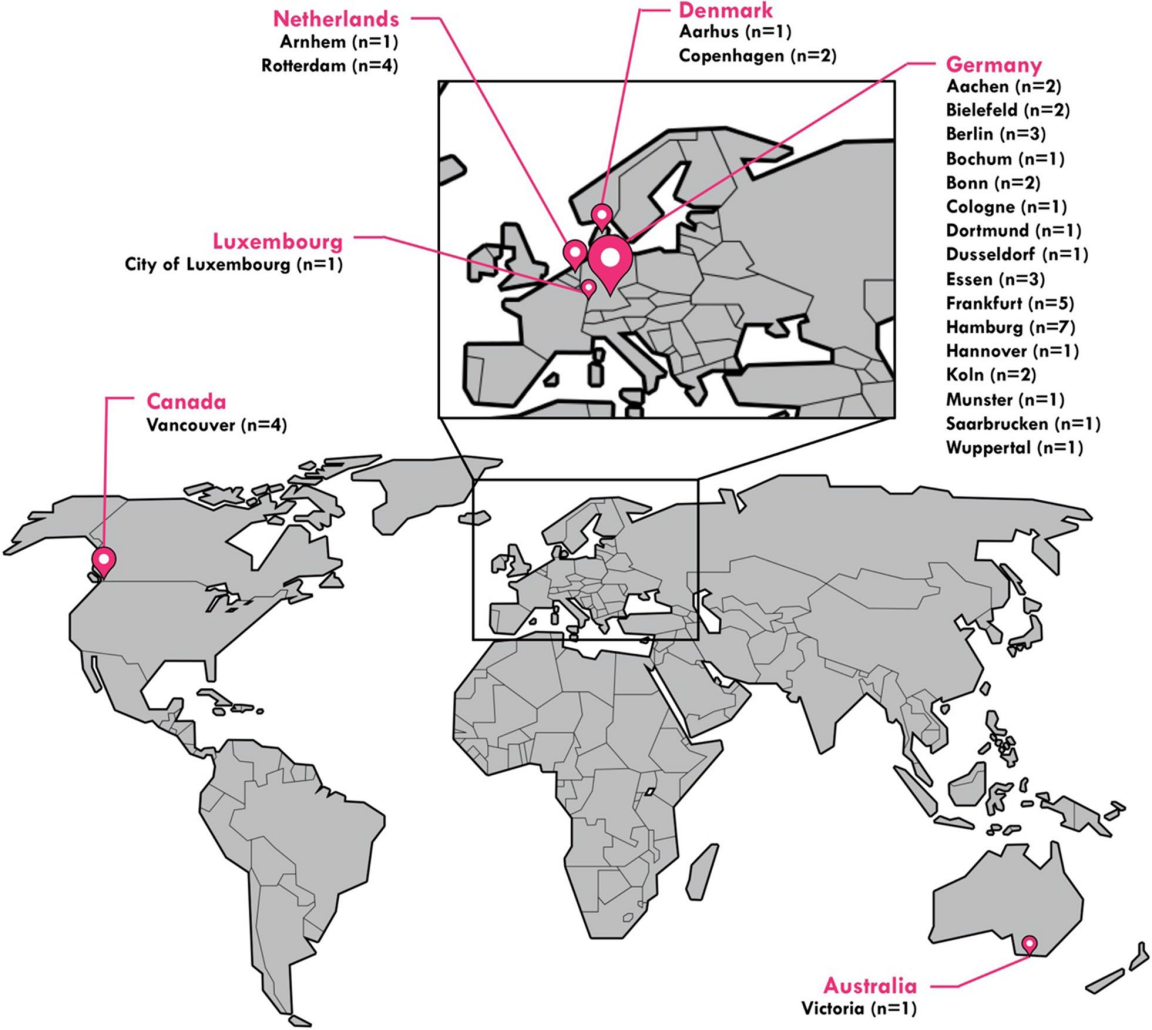
Global distribution of identified SCS that allow non-injection routes of drug consumption (*n* = 48)

Twenty-eight articles identified 48 specific SCS as supervising non-injection routes of consumption (see Fig. 2 for global distribution). The location of each identified SCS is provided in Table 4 by country, city, and service name. Of these SCS, 47 (98%) permitted inhalation, 45 (94%) permitted injection, 12 (25%) permitted intranasal consumption, and no SCS were clearly identified as permitting oral consumption. Note that given the information provided in each article, it was not always possible to identify with certainty whether a specific SCS accommodated a specific mode of drug consumption.

**Table 4:** Location of identified SCS that allow non-injection routes of consumption

Country	City	Name	Citation(s)
Australia	Victoria	Berry Street	[56, 86]
Canada	Vancouver	327 Carrall Street SIS	[79]
		Dr. Peter Centre Residence	[77]
		Pop-up SIS	[87]
		VANDU’s unsanctioned inhalation facility	[15, 16, 41, 87]
Denmark	Aarhus	Aarhus DCR	[36, 78]
	Copenhagen	Maendenes Hjem	[88]
		Skyen	[36, 44, 53, 78]
Germany	Aachen	Drogenhilfe Aachen	[33]
		Suchthlife Aachen	[43]
	Bielefeld	Bielefeld DCR	[45]
		Drogenberatung Bielefeld e.V.	[43]
	Berlin	Birkenstube	[43]
		Fixpunkt	[43]
		SKA	[45]
	Bochum	Krisenhilfe Bochum	[43]
	Bonn	DCR Bonn	[44]
		Verein fur Gefahrdetenhilfe	[43]
	Cologne	Kontaktstelle fur Drogenabhangige	[33]
	Dortmund	KICK—Aide-Hilfe	[43, 45]
	Dusseldorf	Dusseldorfer Drogenhilfe e.V.	[43]
	Essen	Krisenhilfe Essen	[33]
		Suchthilife direkt Essen	[43]
		The Essen DCR	[89]
	Frankfurt	Drogennotdienst Frankfurt	[43]
		Eastside	[43]
		Elbestraße	[50]
		La Strada	[43, 45]
		Nidda 49	[43, 45]
	Hamburg	Abrigado	[33, 38]
		DroBill	[33, 38]
		Drob Inn	[33, 38, 42, 43]
		Fixstern	[33, 38, 42]
		Kodrobs Altona	[43]
		Ragazza e.V.	[33, 38, 43–45]
		Stay Alive	[33, 38, 42, 43]
	Hannover	Fixpunkt/Step gGmbH	[43]
	Koln	KAD I	[43]
		KAD II	[43]
	Munster	Indro	[33, 37, 38, 43]
	Saarbrucken	Drogenhilfezentrum Saarbrucken gGmbH	[43]
	Wuppertal	Gleis 1	[33, 43]
Luxembourg	City of Luxembourg	Abrigado	[1, 46, 90, 91]
Netherlands	Arnhem	Stichting Gelders Centrum Voor Verslavingszorg	[34, 35]
	Rotterdam	Buurthuis	[32]
		Keetje Tippel	[32]
		Moerkerkestraat	[32]
		Pauluskerk	[32, 34]

Of the 48 identified SCS that permitted non-injection routes of consumption, 42 (88%) were considered legally sanctioned, 5 (10%) were identified as unsanctioned, and the legal status for 1 SCS (2%) was not described. The unsanctioned SCS that allowed non-injection routes of consumption were all located in either Australia or Canada, while the sanctioned SCS that allowed alternate routes of consumption were in Europe.

The specific service model was detailed for 45 (94%) of the identified SCS. Forty SCS (89%) were described as integrated models, defined as: “… SCSs [that] are part of larger facilities that offer an array of different services, typically to clients who are unstably housed and/or who inject drugs. Integrated facilities aim to provide comprehensive health and medical care, as well as social services, as a “one-stop-shop” for harm reduction and health care services” [31] (p.14). Three SCS (7%) were identified as ‘specialized’ SCS, which Kerr et al. (n.d.) [31] define as “…a distinct facility that is dedicated to providing SCS. … This type of facility may offer other additional services, such as showers, refreshments, meals, primary care services, counselling, and temporary housing (i.e., shelter). … However, the majority of the facility’s staff time and resources are dedicated to the operations of the SCS program” (p. 13). Two SCS were mobile, which Kerr et al. (n.d.) [31] describe as “…[consisting] of modified vans or buses that contain injection booths and that can be moved to locations where public drug activities occur” (p. 15).

The layout of the SCS was also provided for these 45 SCS (Table 5). Intranasal consumption could occur separately, but often occurred in the inhalation areas or injection spaces. Articles explicitly stated that 6 SCS (13%) allowed visitors to inhale drugs in a separate, often ventilated, room; and 4 SCS (9%) allowed visitors to inhale drugs in an outdoor setting. Articles typically indicated that the SCS included more injection than inhalation spaces; with the exception of literature from the Netherlands which suggested that more spaces for inhalation than injection were provided [32].

**Table 5:** Layout of identified SCS that allow non-injection routes of consumption

Country	Name	Layout	Citation
Australia	Berry Street	Backyard of residential facility	[56]
Canada	327 Carrall Street SIS	Storefront space with a back room with two small tables divided by a temporary wall for injection and washroom; after four months, visitors were allowed to consume via inhalation in the washroom with a fan	[79]
	Dr. Peter Centre Residence	Injection within residents' rooms and inhalation accommodated in an outside area	[77]
	Pop-up SIS	Tables within a tent	[87]
	VANDU’s unsanctioned inhalation facility	Bathroom with ventilation system	[15]
		Inhalation room with fan accessible to one person at a time	[41]
		Storefront location with a lobby, front desk area, injection room, and small adapted washroom with ventilation system accessible to one person at a time	[16]
Denmark	Aarhus DCR	5 spaces for injection and 2 spaces for inhalation	[78]
		Injection room, smoking room, health clinic	[36]
	Maendenes Hjem	Various rooms for different purposes including an injection and smoking room	[88]
	Skyen	Spaces for injection and inhalation (air conditioned)	[53]
		8 spaces for injection and 6 spaces for inhalation	[78]
		7 spaces for inhalation	[44]
		Booths for injection and a separate room for inhalation	[36]
Germany	Abrigado	4 spaces for injection and 4 spaces for inhalation	[38]
		10 rooms for different purposes; 4 spaces for injection and 4 spaces for inhalation	[33]
	Birkenstube	6 spaces for injection/intranasal and 4 spaces for inhalation	[43]
	DCR Bonn	Ground floor: care area with lounge and kitchen First floor: counselling centre for drug use Second floor: medical clinic (outpatient) Third floor: crisis intervention (inpatient; short term; maximum 6 clients) Backyard: 5 spaces for injection and 3 spaces for inhalation	[44]
	Drob Inn	10 spaces for injection and 5 spaces for inhalation/intranasal	[43]
		7 spaces for injection and 3 spaces for inhalation	[38]
		15 rooms for different purposes; 7 spaces for injection and 3 spaces for inhalation	[33]
		7 spaces for injection and 3 spaces for inhalation	[42]
	DroBill	6 spaces for injection and 1 space for inhalation	[38]
		8 rooms for different purposes; 8 spaces for injection and 1 space for inhalation	[33]
	Drogenberatung Bielefeld e.V.	8 spaces for injection/intranasal and 8 spaces for inhalation	[43]
	Drogenhilfe Aachen	~ 14 rooms for different purposes; 2 spaces for injection and 2 spaces for inhalation	[33]
	Drogenhilfezentrum Saarbrücken gGmbH	13 spaces for injection/intranasal and 3 spaces for inhalation	[43]
	Drogennotdienst Frankfurt	10 spaces for injection/intranasal and 5 spaces for inhalation	[43]
	Düsseldorfer Drogenhilfe e.V.	6 spaces for injection/intranasal and 3 spaces for inhalation/intranasal	[43]
	Eastside	8 spaces for injection/intranasal; 2 of those spaces are for inhalation	[43]
	Fixpunkt	2 mobile consumption rooms; 3 spaces for injection/intranasal	[43]
	Fixpunkt/Step gGmbH	9 spaces for injection and 3 spaces for intranasal	[43]
	Fixstern	6 spaces for injection and 3 spaces for inhalation	[38]
		~ 10 rooms for different purposes; 6 spaces for injection and 3 spaces for inhalation	[33]
		6 spaces for injection and 3 spaces for inhalation	[42]
	Gleis 1	5 spaces for injection and 4 spaces for inhalation	[43]
		~ 16 rooms for different purposes; 5 spaces for injection and 3 spaces for inhalation	[33]
	Indro	4 spaces for injection/intranasal and 1 space for inhalation	[43]
		6 spaces for injection (no booth-like partitions, only 4 participants at a time) and one booth with air exhaust for inhalation	[37]
		4 spaces for injection (maximum 6 participants) and 1 space for inhalation	[38]
		~ 9 rooms for different purposes; 4–6 spaces for injection and 1 space for inhalation	[33]
	KAD I	3 spaces for injection/intranasal/inhalation	[43]
	KAD II	6 spaces for injection/intranasal and 2 spaces for inhalation	[43]
	KICK—Aide-Hilfe	8 spaces for injection and 8 spaces for inhalation/intranasal	[43]
	Kodrobs Altona	4 spaces for injection and 2 spaces for inhalation/intranasal	[43]
	Kontaktstelle für Drogenabhängige	~ 14 rooms for different purposes; 2 spaces for injection and 1 space for inhalation; smallest facility in Germany at the time	[33]
	Krisenhilfe Bochum	5 spaces for injection/intranasal and 3 spaces for inhalation	[43]
	Krisenhilfe Essen	~30 rooms for different purposes; 8 spaces for injection and 4 spaces for inhalation	[33]
	La Strada	7 spaces for injection/intranasal	[43]
	Nidda 49	12 spaces for injection/intranasal/inhalation	[43]
	Ragazza e.V.	4 spaces for injection/intranasal and 6 spaces for inhalation (8 parallel consumptions maximum)	[43]
		6 spaces for injection and 2 spaces for inhalation	[38]
		10 rooms for different purposes; 5 spaces for injection and 3 spaces for inhalation	[33]
	Stay Alive	8 spaces for injection, inhalation, and intranasal	[43]
		6 spaces for injection and 2 spaces for inhalation	[38]
		~ 13 rooms for different purposes; 6 spaces for injection and 2 spaces for inhalation	[33]
		6 spaces for injection and 2 spaces for inhalation	[42]
	Suchthilfe direkt Essen	8 spaces for injection/intranasal and 5 spaces for inhalation	[43]
	Suchthlife Aachen	5 spaces for injection; 2 of which are for inhalation	[43]
	The Essen DCR	8 spaces for injection and 4 spaces for inhalation	[89]
	Verein für Gefährdetenhilfe	5 spaces for injection/intranasal and 3 spaces for inhalation	[43]
Luxembourg	Abrigado	Injection room and inhalation room (which opened in 2012)	[90]
		Injection room with 8 tables and inhalation room with 6 tables	[46]
		7 spaces for injection and 3–4 spaces for inhalation (pilot project; February 2012)	[1]
		Supervised injection and inhalation room	[91]
Netherlands	Buurthuis	5–6 spaces for injection and 10–14 spaces for inhalation	[32]
	Keetje Tippel	5 spaces for injection and 14 spaces for inhalation	[32]
	Moerkerkestraat	5–6 spaces for injection and 10–14 spaces for inhalation	[32]
	Pauluskerk	Various rooms for different purposes; 1 space for injection (2 tables for 6 participants at a time) and 1 space for inhalation (each space ~ 3 × 5 m)	[34]
		20 spaces for injection and 20 spaces for inhalation	[32]
	Stichting Gelders Centrum Voor Verslavingszorg	Entry controlled by worker in a small office inside the front door; large open sitting/recreation area space (10 × 8 m); 1 space for injection and 1 space for inhalation regulated by staff member (both rooms 2 × 3 m; 8 people at a time; 1 m square window in doors for observation)	[34]

Articles provided the hours of operation for half (*n* = 24; 50%) of the identified SCS that permit non-injection routes of consumption, with hours ranging from as few as 3 h per day to as many as 24 h per day. While some SCS were only open on select days, others were open seven days a week. Operational hours were often dependent on funding and the staffing complement. In total, articles provided details on staffing for 41 (85%) of the identified SCS that permit alternate routes of consumption. Of these 41 SCS, 35 (85%) discussed employing healthcare professionals (e.g., nurses, physicians), 16 (39%) discussed employing social workers, 27 (66%) discussed employing counsellors, 2 (5%) discussed employing peer workers (“…members of the community who have experience in safe illicit drug practices” [15]), and 35 (85%) discussed employing other staff (e.g., outreach workers, security guards, volunteers). Both of the SCS that employ peer workers were in Canada.

Thirty-eight (79%) of the identified SCS provided eligibility criteria for entry into SCS. Common eligibility criteria included a minimum age for entry (*n* = 27; 71%), having an active or long history of illegal drug use (i.e., not consuming illegal drugs for the first time; *n* = 32; 84%), and not being intoxicated at the time of entry (*n* = 15; 39%). Half of the identified SCS did not allow people who were on opioid agonist treatment to use the SCS (*n* = 19; 50%); however, in some cases conflicting information regarding this restriction was provided. Other eligibility criteria included not allowing people who were pregnant to consume drugs in the SCS (*n* = 3; 8%), and only allowing people who lived in the city of the SCS to use the site (*n* = 3; 8%). Notably, literature on the Krisenhilfe Essen SCS (Germany) indicated that it allowed people from out of town who were experiencing withdrawal to use the site on a one time basis to alleviate their symptoms [33]. Two facilities (Pauluskerk and Stichting Gelders Centrum Voor Verslavingszorg in the Netherlands) require program participants to be pre-approved to use the site [34, 35]. Other rules beyond eligibility criteria included restrictions on: drug sharing (*n* = 31; 82%); reusing equipment (*n* = 27; 71%); the substances that could be consumed within the SCS (*n* = 8; 21%); and the route that substances could be consumed (*n* = 4; 11%). Further, some facilities had rules prohibiting violence towards staff or other people within the SCS (*n* = 9; 24%), or banning peer assistance for drug consumption (*n* = 9; 24%).

Articles provided information on the time limits imposed on people using the consumption room for 14 of the identified SCS (37%). These time limits most commonly ranged from 5 to 30 min in length (*n* = 8; 57%). Alternatively, articles that discussed the Aarhus DCR (Denmark), Buurthuis (Netherlands), and Moerkerkestraat (Netherlands) indicated that there were no time limits for consumption [32, 36], while in Pauluskerk (Netherlands) there was a time limit for inhalation but not for injection [32]. Pauluskerk (Netherlands), Keetje Tippel (Netherlands), and Indro (Germany) all had different time limits for inhalation compared to injection, with shorter time limits imposed on those inhaling their drugs [32, 37]. Finally, Stoever [38] stated that Abrigado, Ragazza e.V., and Drobill in Germany enforced a time limit in the consumption rooms without specifying the length.

Articles discussed the services provided at 45 of the identified SCS (94%). Forty-two (93%) SCS provided social services (e.g., referrals to substance use disorder treatment programs); 42 (93%) provided health services (e.g., HIV testing, medical treatment); 36 (80%) distributed harm reduction supplies (e.g., needle exchange, condoms); 23 (51%) provided basic needs (e.g., food, showers, laundry); 36 (80%) provided education (e.g., safer use counselling, health education); 10 (22%) provided shelter (e.g., overnight accommodations); and 25 (56%) provided other services (e.g., drop-ins, referrals to further services) to program participants. However, one article discussed how the ability to provide services such as education was reduced in inhalation areas compared to the injection spaces, due to potential air quality-related occupational health and safety concerns for staff [36].

These articles further discussed the number of program participants for 20 (42%) of the identified SCS. In general, the number of daily program participants varied depending on where the SCS was located; SCS located within a concentrated drug scene typically had a higher number of people accessing the site than SCS that were located away from areas where people who use drugs tended to spend their time [27, 39]. The number of daily program participants also varied depending on other contextual and site characteristics [40] such as the target population, service hours, and capacity of each SCS.

As shown in Table 6, only 4 articles (8%) provided operating costs for specific SCS that allow alternative routes of consumption. Annual costs ranged from $97,203.00 CAD ($108,311.06 USD 2020) per year for the unsanctioned inhalation facility run by Vancouver Area Network of Drug Users in Canada [41] to ƒ1,200,000.00 NLG ($1,163,954.24 USD 2020) per year for Pauluskerk in the Netherlands [34]. All available data extracted for each of these variables are provided for each SCS in Additional file 5.

**Table 6:** Operational costs of SCS that allow non-injection routes of consumption

Name (location)	Location	Operational costs as reported	Annual operational costs in USD^a^	Citation
DCR Bonn, Ragazza, and Skyen	Bonn and Hamburg, Germany Copenhagen, Denmark	Cost per participant in mobile SCS higher than fixed-site SCS (less visits per day yet require similar staffing levels)	NA	[44]
Indro	Munster, Germany	Annual cost: €125,000.00	$187,488.11^b^	[37]
Pauluskerk	Rotterdam, Netherlands	Annual budget: ƒ1,200,000.00	$1,163,954.24^c^	[34]
Unsanctioned inhalation facility	Vancouver, Canada	Annual volunteer stipends: $47,203.00 CAD	Annual volunteer stipends: $64,068.74^d^	[41]
		Annual rent and drug use equipment: $50,000.00 CAD	Annual rent and drug use equipment: $55,713.91^d^	
		Total annual cost: $97,203.00 CAD	Total annual cost: $108,311.06^d^	

*NA* not applicable

^a^Exchange rates based on the first day of the month in which the article was published and then adjusted for inflation in 2020

^b^1 Euro = 1.07640 USD (2003); $134,550.00 USD (2003)

^c^1 NLG = 0.54666 USD (1993); $655,988.00 USD (1993)

^d^1 CAD = 0.82095 USD (2004); annual volunteer stipend: $38,751.40 USD (2004); annual rent and drug use equipment: $41,047.60 USD (2004); total annual cost: $79,798.90 USD (2004)

### Characteristics of program participants

Twenty-one articles (26%) provided demographic or other data on people who access SCS that permit non-injection routes of consumption in general (without necessarily identifying the SCS by name as incorporating non-injection routes of consumption). Some SCS targeted structurally vulnerable populations: Fixstern, Stay Alive, and Drob Inn in Germany targeted their services toward people who consumed drugs in public [42]; Ragazza e.V. (Germany) [33, 43–45] and Keetje Tippel (Netherlands) [32] targeted their services towards women, particularly sex workers; and Buurthuis and Moerkerkestraat in the Netherlands targeted people experiencing homelessness [32].

Thirteen articles (62%) provided data on the age of program participants, and 4 articles (19%) provided data on length of drug use history. People who use SCS that allow alternate routes of consumption were more likely to be 30 years of age or older [1, 34, 39, 40, 46–52], and to have used drugs for at least 10 years [39, 40, 51]. In addition, Zobel and Dubois-Arber (2004) noted that people who used the inhalation rooms in Switzerland had a long history of drug use, and included people who had previously consumed their drugs through injection as well as those who had never injected their drugs [52].

Thirteen articles (62%) provided information on the sex and/or gender of the program participants. Most of the sites reported seeing more men than women [1, 32, 34, 37, 40, 43, 46–51, 53] with two exceptions: (1) Keetje Tippel in the Netherlands served women who participated in sex work and only allowed women to access the SCS [32]; and (2) the 12 SCS in Germany had a higher proportion of women than men in the 18–25 year old age group between 2001 and 2009 [43].

Six articles (29%) provided information on the ethnicity of the program participants of SCS that allow non-injection routes of consumption. The articles reported different kinds of information across the sites, making it difficult to synthesize. For example, the program participants of four SCS in the Netherlands (Pauluskerk, Keetje Tippel, Buurthuis, and Moerkerkestraat) were broken down into Dutch (45%) and other (55%) [32], whereas Hunt [54] stated that SCS in the Netherlands, Germany, and Switzerland saw “[a] large proportion of service users [who] appeared to come from minority ethnic groups…” (p. 10).

Ten articles (48%) provided information on the housing status of program participants. Overall, a disproportionately large amount of participants were currently or previously living in unstable housing situations [32, 34, 39, 40, 48, 50, 53, 55], although the proportion varied by site and location. There were two notable exceptions to this trend: (1) the program participants of the inhalation room in Biel/Bienne, Switzerland did not experience homelessness [52], and (2) all participants of the SCS at Berry Street in Victoria, Australia were residents of the facility [56]. See Additional file 6 for more detailed information on the program participant characteristics provided by each of the included articles.

## Discussion

We conducted the first systematic search and synthesis of academic and grey literature on SCS that permit non-injection routes of consumption. We identified 193 articles that discussed SCS allowing alternate routes of consumption in some capacity, and ultimately included 39 articles which identified 48 SCS that permit program participants to consume orally, intranasally, or via inhalation.

While systematic reviews of SCS have been conducted in the past [3, 4, 57], our study is the first to focus explicitly on what is known regarding the service models of SCS that accommodate drug consumption by oral, intranasal, or inhalation routes. Despite capturing 193 potentially relevant articles that discussed supervising non-injection forms of drug consumption, only 39 identified specific examples of SCS allowing alternate routes of consumption and/or provided program participant characteristics for people who use drugs orally, intranasally, or by inhalation. This indicates that there is limited published literature focusing specifically on this type of SCS. Some characteristics (e.g., service provision, rules and eligibility, staffing, hours, layout) are more clearly documented in the available literature than others (e.g., number of visitors, target population, operational costs), and the majority of identified SCS were located in one country (Germany; 71%).

According to available literature, program participants were typically men over the age of 30 and part of a structurally vulnerable population (e.g., experiencing homelessness or unstable housing). This is consistent with characteristics of program participants reported in research on supervised injection services [4]. The range of site characteristics identified in our review are also largely consistent with the literature on supervised injection services (see the Medically Supervised Injecting Centre in Australia [58] for an example of a supervised injection service), which is unsurprising given the majority of identified SCS also served people who inject drugs. For example, the majority of SCS identified in this scoping review were legally sanctioned, integrated models with comparable eligibility criteria (e.g., minimum age, history of illegal drug use) and service provision (e.g., social services, harm reduction supplies, education) to supervised injection services. Notable differences between the SCS in this study compared to supervised injection services included alternative layouts to accommodate spaces for non-injection routes of consumption, primarily inhalation (i.e., ventilated rooms, outdoor areas), and shorter time limits for inhalation compared to injection. Longer time limits for injection may reflect the more extensive and time consuming drug preparation process required compared to consumption via inhalation [59]. Ultimately, it appears that integrating non-injection routes of consumption within supervised injection services does not require increased resources to implement or operate the non-injection portion of the service, beyond physical infrastructure requirements for accommodating inhalation.

Increasingly, SCS are incorporating services to address the risks associated with consuming adulterated drugs from the toxic drug supply. These services include incorporating drug checking services [60, 61] and the provision of pharmaceutical grade alternatives to street drugs (e.g., safe supply) [62, 63]; although these services are largely targeted to people who use opioids and often do not address the needs of people who use stimulants [64]. As these services are relatively recent developments, they were not discussed in the included articles and therefore the extent to which they are incorporated within SCS that allow non-injection routes of consumption remains unclear. However, the current emphasis on innovative solutions to the overdose crisis [65] highlights the need for SCS to be responsive to the needs of their participants. Furthermore, the current COVID-19 pandemic has also demonstrated the importance of flexibility in response to the evolving needs of SCS participants [66]. People who smoke illegal drugs may be particularly at risk for complications associated with respiratory illness [67]. Many people who use drugs have been impacted by sudden closures of their SCS due to their inability to meet public health directives [68, 69], while other SCS had to reduce their capacity to meet physical distancing requirements [70]. The operational characteristics of both injection and non-injection SCS should be flexible and continuously adapted to address local needs and context.

We found a range of rules and criteria across the different SCS. Many SCS enacted eligibility criteria that risk excluding the populations most in need of the service. For example, women who use drugs are more likely to experience violence and are at a higher risk of blood-borne virus infections [71], yet some SCS policies (e.g., excluding people who are pregnant or who have children with them), disproportionately impact women attempting to access SCS. Many SCS did not permit people who are younger than 18 years of age to consume drugs on site. This is problematic because youth who consume illegal drugs are at heightened risk of hazardous consumption patterns or other activities (e.g., sex work) that increase risk of exposure to blood-borne viruses [72, 73]. Excluding people who wish to access these services on the basis of age does not take into account their needs [74, 75], and eliminates an opportunity to improve health outcomes amongst an especially vulnerable subpopulation of people who use drugs. Furthermore, SCS typically have limited operating hours, commonly operating only during the day. Recent research indicates that program participants and consumption patterns vary according to the time of day, with an increase in vulnerable populations (e.g., women and those experiencing homelessness) and stimulant use during night hours [76]. Restricting operating hours may also increase harms to vulnerable subpopulations of people who use drugs (e.g., violence, overdose). In contrast, several SCS sought to address these service gaps by specifically targeting populations of women, including those who engage in sex work [32, 33, 44], permitting people under 18 to access the SCS [33], operating 23–24 h per day [36, 77, 78], or extending hours past midnight [32, 33, 79].

The level of reported detail on SCS varies considerably by jurisdiction, and appears to be impacted by political climates [80]. In recent years, an increasing number of Canadian SCS permitted intranasal and oral consumption [81], and Lethbridge, Alberta opened the first SCS in North America to permit all routes of consumption (injection, inhalation, intranasal, oral) in February 2018 [17]. However, limited data has been published on these SCS to date [82]. This contrasts sharply with the implementation of Canada’s first SCS in 2003, which was contingent on extensive research exploring its impact [83]. In Europe, SCS implementation was much less controversial than in Canada, which generally resulted in smaller investment in evaluations of European SCS [40]. For example, although documentation and evaluation are legally required to operate an SCS in Germany, many of the early reports were to funders and focused on quality improvement rather than peer-reviewed evaluation of the sites [27]. This de-emphasis on research resulted in only 41% of the included articles being peer-reviewed, and over half of the articles being descriptive (i.e., did not systematically collect and analyze qualitative and/or quantitative data). While the provision of SCS should not be contingent on scientific evaluation, rigorous research on the range of extant models is essential to facilitate knowledge translation among other researchers and policy makers to ensure future implementation efforts are as effective as possible.

Adding to the lack of clarity in the literature, many terms to denote SCS were used in the included articles, often interchangeably, despite some of the terms having slightly different implications (e.g., different routes of consumption, different levels of service provision). The use of these many different terms also has implications for the level of support offered by the community [84]. Implementing a common nomenclature for reporting on SCS models and program participant characteristics should include a commitment to accurate terminology to describe these services, and may facilitate knowledge translation to members of the community, policy and decision makers, and other stakeholders.

We recommend standardizing conventions for describing SCS site and program participant characteristics to facilitate reporting and comparisons, and ensure the range of existing models is accurately represented. In particular, future survey research that aims to construct a census of existing service models, using standardized definitions to outline characteristics and describe program participant populations would greatly advance understanding of supervised non-injection drug consumption service models. It would also support those seeking to implement SCS in determining the site characteristics most appropriate for their specific context and potential trade-offs with choosing one type of model over another. Furthermore, a clear description of the site characteristics is necessary to systematically evaluate whether these SCS are meeting their goals. The characteristics of people who use SCS depend in part on the target population, eligibility criteria, and layout of the SCS, and providing an aggregate summary of typical program participants of SCS may not be a useful measure despite the common aim to reduce harm to marginalized populations who are consuming drugs [3]. Instead, it may be more important to assess how well the site characteristics of each SCS are contributing to that site’s overall objectives. In particular, future research should examine the impacts of SCS policies, and explore whether these policies inadvertently exacerbate inequities between different subpopulations of people who use drugs.

### Limitations

This scoping review is subject to limitations, the first of which was the exclusion of articles that were not published in English. Many SCS that permit non-injection routes of consumption exist in Europe, and only including articles in English may have resulted in the exclusion of some relevant literature [57]. However, of the 807 full-text articles we screened from the peer-reviewed databases, only 29 (3.6%) were excluded because they were not available in English. This is consistent with other systematic reviews of supervised injection services [4], and the preponderance of information on SCS from predominantly English-speaking countries were also observed in reviews that did not limit their inclusion criteria to English language studies [3]. In addition, the majority of our included articles discussed SCS in Europe, including those located in countries where English is not the primary language spoken. Second, the nature of our study (a review of previously published literature) meant we were not able to reconcile inconsistent reporting between articles, which limits the clarity of site and program participant characteristics reported here. This is especially important as much of the data provided by the included articles were unclear; many articles discussed SCS characteristics in aggregate, did not specify which characteristics apply to each SCS, and/or provided conflicting information. However, this lack of clarity highlights the lack of standardized reporting of SCS and the need for more consistency moving forward. Third, while we identified 48 SCS in 6 countries as allowing alternate routes of consumption, this may be an underestimation of the diversity and total number of SCS that permit non-injection routes of consumption globally. We only report on SCS which are documented or described in the peer-reviewed and grey literature; not all sites are documented in the literature, sites may have evolved or changed since publication, and more sites have been implemented since we conducted the search for this scoping review, particularly in Canada [81]. Finally, while no formal quality assessment is required for scoping reviews [85], not assessing the quality of the data provided by the articles may have placed inadvertent emphasis on lower quality data. Despite these limitations, our review is the first to comprehensively capture the existing data on SCS that permit non-injection routes of consumption and to recommend improvements to the current knowledge base.

## Conclusion

Numerous articles discussed SCS that permit non-injection routes of consumption. Overall, the level of detail available regarding these sites is quite low, with inconsistent and imprecise reporting of the routes of consumption permitted, the site characteristics, and the characteristics of program participants of each site. While the evidence documenting the provision of supervised injection services is robust, there is comparatively limited literature discussing sites that allow alternate routes of consumption. Clear knowledge of existing SCS models that permit non-injection routes of consumption will facilitate further innovation, implementation, and evaluation of these services across jurisdictions.

## Supplementary information

**Additional file 1.** All search strategies for databases not presented in the body of the manuscript.

**Additional file 2.** A list of all terms used in the grey literature search strategy.

**Additional file 3.** List of exemplar articles used to verify and test the search strategy.

**Additional file 4.** Full details of the screening process including grey literature screening, external reference list screening, and screening for reference list of included articles.

**Additional file 5.** Data extraction of SCS characteristics.

**Additional file 6.** Data extraction of program participant characteristics.

## Data Availability

All data generated or analysed during this study are included in this published article and its supplementary files. Additional information may be provided upon reasonable request to the corresponding author.

## Abbreviations

CAD: Canadian dollar
HIV: Human immunodeficiency virus
NLG: Dutch guilder
PRISMA-ScR: PRISMA extension for scoping reviews
SCS: Supervised consumption services
USD: American dollar
