# Supplementary material for: To what extent do supervised drug consumption services incorporate non-injection routes of administration? A systematic scoping review documenting existing facilities
Source: Harm Reduct J. 2020 Oct 7;17:72. doi: 10.1186/s12954-020-00414-y (PMC7539556; doi:10.1186/s12954-020-00414-y)
Supplement: Supplementary file 1 — Additional file 1. All search strategies for databases not presented in the body of the manuscript. [file 12954_2020_414_MOESM1_ESM.docx]

**Additional file 1 Search strategies**

**Database: Ovid MEDLINE(R) Epub Ahead of Print, In-Process & Other Non-Indexed Citations, Ovid MEDLINE(R) Daily and Ovid
MEDLINE(R) <1946 to August 07, 2020^[[1]](#footnote-1)^>
Search Strategy:
--------------------------------------------------------------------------------**

| **#** | **Search Statement** | **Results** |
| --- | --- | --- |
| 1 | ((supervised or safe or safer) adj (injection or injecting or inhalation or inhaling or smoking or snorting or intranasal)).mp. | 787 |
| 2 | "drug consumption room"/ or ((injection or injecting or inhalation or inhaling or consumption or smoking or snorting or intranasal) adj (room or rooms or facility or facilities or service* or center or centers or centre or centres)).mp. | 816 |
| 3 | (((safe* or supervised) adj1 consumption) and (drug or drugs or opioid* or addict* or harm reduction or overdose*)).mp. | 150 |
| 4 | "fixing room*".mp. | 1 |
| 5 | "overdose prevention site*".mp. | 19 |
| 6 | 1 or 2 or 3 or 4 or 5 | 1,430 |
| 7 | (airport or airports or operating room* or alcohol consump*).mp. or exp Operating Rooms/ or exp Alcohol Drinking/ or exp Anaesthesia/ or exp Ophthalmology/ or *"Needlestick Injuries"/ or *Smoking/ or *Vaccines/ | 217,614 |
| 8 | (ecigarette* or e-cigarette or cigarette smoking or (smoking adj2 cessation) or secondhand smok* or second handsmok* or "stop smoking" or vaccin* or immuniz* or operat* room* or surgical).mp. | 1,914,462 |
| 9 | 7 or 8 | 2,043,582 |
| 10 | 6 not 9 | 1,006 |
| 11 | remove duplicates from 10 | 990 |
| 12 | limit 11 to animals | 61 |
| 13 | limit 12 to humans | 19 |
| 14 | 12 not 13 | 42 |
| 15 | 11 not 14 | 948 |
| 16 | limit 15 to dt=20170101-20170912 | 49 |
| 17 | 15 | 948 |
| 18 | limit 17 to yr="1860 - 2016" | 642 |
| 19 | 16 or 18 | 690 |

**Database: Embase <1974 to 2020 August 07>
Search Strategy:
--------------------------------------------------------------------------------**

| **#** | **Search Statement** | **Results** |
| --- | --- | --- |
| 1 | ((supervised or safe or safer) adj (injection or injecting or inhalation or inhaling or smoking or snorting or intranasal)).mp. | 971 |
| 2 | "drug consumption room"/ or ((injection or injecting or inhalation or inhaling or consumption or smoking or snorting or intranasal) adj (room or rooms or facility or facilities or service* or center or centers or centre or centres)).mp. | 1,085 |
| 3 | (((safe* or supervised) adj1 consumption) and (drug or drugs or opioid* or addict* or harm reduction or overdose*)).mp. | 269 |
| 4 | "fixing room*".mp. | 2 |
| 5 | "overdose prevention site*".mp. | 18 |
| 6 | 1 or 2 or 3 or 4 or 5 | 1,918 |
| 7 | (airport or airports or operating room* or alcohol consump*).mp. or exp Operating Rooms/ or exp Alcohol Drinking/ or exp anaesthesia/ or exp ophthalmology/ or *"Needlestick Injuries"/ or *Smoking/ or *Vaccines/ | 666,400 |
| 8 | (ecigarette* or e-cigarette or cigarette smoking or (smoking adj2 cessation) or secondhand smok* or second hand smok* or "stop smoking" or vaccin* or immuniz* or operat* room* or surgical).mp. | 2,386,988 |
| 9 | 7 or 8 | 2,864,241 |
| 10 | 6 not 9 | 1,289 |
| 11 | remove duplicates from 10 | 1,277 |
| 12 | limit 11 to animals | 67 |
| 13 | 11 not 12 | 1,210 |
| 14 | limit 13 to yr="1883 - 2016" | 852 |
| 15 | limit 13 to dd=20170101-20170912 | 49 |
| 16 | limit 13 to rd=20170101-20170912 | 17 |
| 17 | 14 or 15 or 16 | 878 |

**Database: PsycINFO <1806 to August Week 1 2020>
Search Strategy:
--------------------------------------------------------------------------------**

| **#** | **Search Statement** | **Results** |
| --- | --- | --- |
| 1 | ((supervised or safe or safer) adj (injection or injecting or inhalation or inhaling or smoking or snorting or intranasal)).mp. | 407 |
| 2 | "drug consumption room"/ or ((injection or injecting or inhalation or inhaling or consumption or smoking or snorting or intranasal) adj (room or rooms or facility or facilities or service* or center or centers or centre or centres)).mp. | 429 |
| 3 | (((safe* or supervised) adj1 consumption) and (drug or drugs or opioid* or addict* or harm reduction or overdose*)).mp. | 75 |
| 4 | "fixing room*".mp. | 1 |
| 5 | "overdose prevention site*".mp. | 7 |
| 6 | 1 or 2 or 3 or 4 or 5 | 684 |
| 7 | (airport or airports or operating room* or alcohol consump*).mp. or exp Operating Rooms/ or exp Alcohol Drinking/ or exp anaesthesia/ or exp ophthalmology/ or *"Needlestick Injuries"/ or *Smoking/ or *Vaccines/ | 21,573 |
| 8 | (ecigarette* or e-cigarette or cigarette smoking or (smoking adj2 cessation) or secondhand smok* or second hand smok* or "stop smoking" or vaccin* or immuniz* or operat* room* or surgical).mp. | 55,358 |
| 9 | 7 or 8 | 75,544 |
| 10 | 6 not 9 | 527 |
| 11 | remove duplicates from 10 | 526 |
| 12 | limit 11 to animal | 3 |
| 13 | 11 not 12 | 523 |
| 14 | limit 13 to yr="1860 - 2016" | 382 |
| 15 | limit 13 to up=20170101-20170912 | 30 |
| 16 | 14 or 15 | 408 |

**CINAHL**  Searched August 9, 2020 limited to September 2017

| **#** | **Query** | **Limiters/Expanders** | **Results** |
| --- | --- | --- | --- |
| S1 | (supervised or safe or safer) N1 (injection or injecting or inhalation or inhaling or smoking or snorting or intranasal) | Search modes - Find all my search terms | 683 |
| S2 | (injection or injecting or inhalation or inhaling or smoking or snorting or intranasal) N1 ( room or rooms or facility or facilities or service* or center or centers or centre or centres) | Search modes - Find all my search terms | 1,151 |
| S3 | ((safe* or supervised) n1 consumption) AND ((drug or drugs or opioid or addict* or harm reduction or overdose*)) | Search modes - Find all my search terms | 139 |
| S4 | "fixing room" | Search modes - Find all my search terms | 12 |
| S5 | "overdose prevention site*" | Search modes - Find all my search terms | 18 |
| S6 | S1 OR S2 OR S3 OR S4 OR S5 | Search modes - Find all my search terms | 1,727 |
| S7 | airport or airports or "operating room*" or "alcohol consumption" | Search modes - Find all my search terms | 34,343 |
| S8 | (MH "Operating Rooms") | Search modes - Find all my search terms | 9,021 |
| S9 | (MH "Alcohol Drinking+") | Search modes - Find all my search terms | 30,952 |
| S10 | (MH "Anesthesia+") | Search modes - Find all my search terms | 47,330 |
| S11 | (MH "Needlestick Injuries") | Search modes - Find all my search terms | 3,941 |
| S12 | (MH "Smoking+") | Search modes - Find all my search terms | 71,603 |
| S13 | (MH "Vaccines+") | Search modes - Find all my search terms | 46,365 |
| S14 | (MH "Immunization+") | Search modes - Find all my search terms | 27,527 |
| S15 | (ecigarette* or e-cigarette or cigarette smoking or (smoking N2 cessation) or second hand smok* or secondhand smok* or "stop smoking" or vaccin* or immuniz* or operat* room* or surgical) | Search modes - Find all my search terms | 367,707 |
| S16 | S7 OR S8 OR S9 OR S10 OR S11 OR S12 OR S13 OR S14 OR S15 | Search modes - Find all my search terms | 480,420 |
| S17 | s6 not s16 | Search modes - Find all my search terms | 825 |
| S18 | EM 19000101 - 20170912 | Search modes - Find all my search terms | 5,668,896 |
| S19 | S17 AND S18 | Search modes - Find all my search terms | 537 |

**PROSPERO** Searched September 12, 2017 Results =3


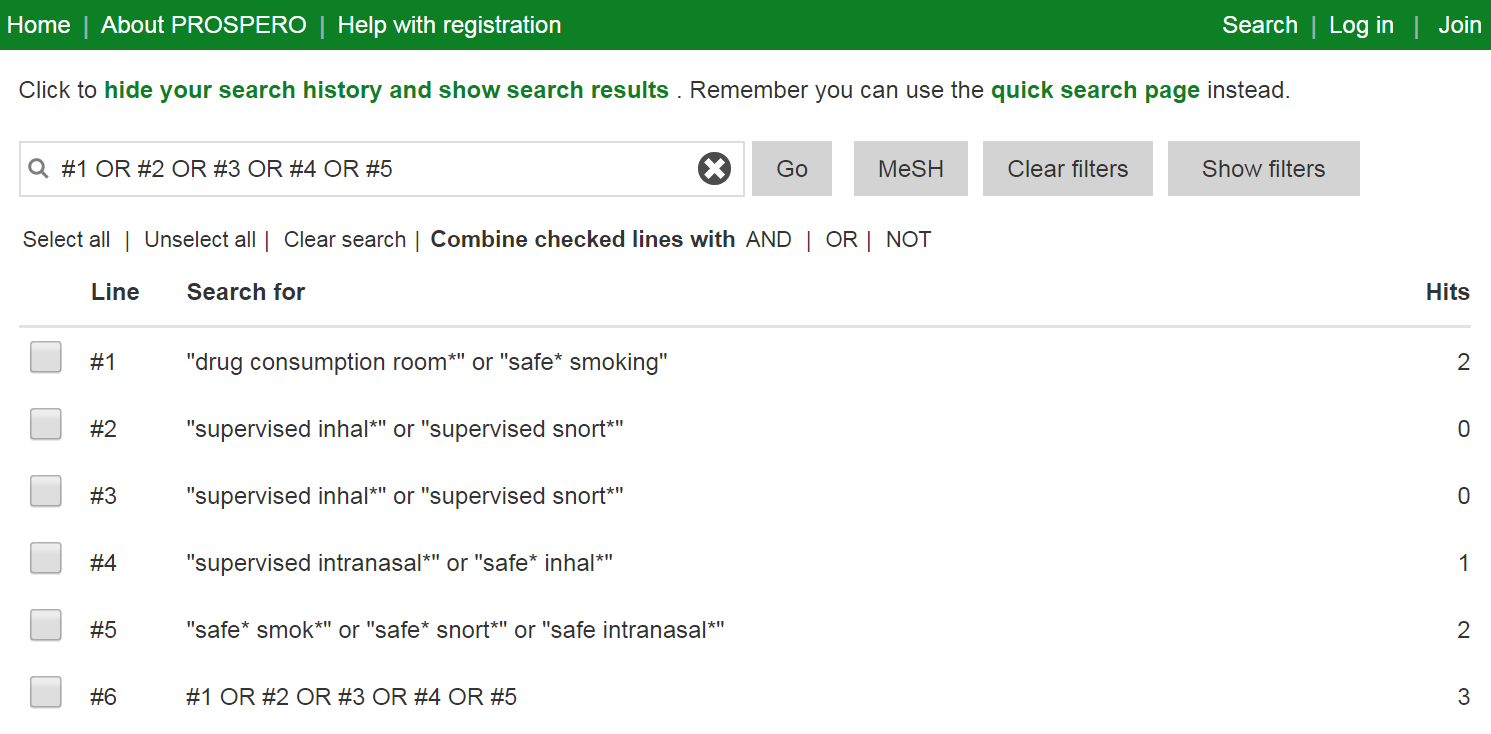


**Proquest Dissertations and Theses Global** Searched September 12 = 52 references

all("drug consumption" NEAR/2 (room* OR unit* OR facilit* OR galler*)) OR all((supervise* OR safe OR safer) NEAR/1 (smok* OR inhal* OR snort* OR intranasal* or inject*))

**Web of Science** Timespan: All years. Indexes: SCI-EXPANDED, SSCI, A&HCI, CPCI-S, CPCI-SSH, BKCI-S, BKCI-SSH, ESCI, CCR-EXPANDED, IC. Searched September 12, 2017 = 2 references

TOPIC: (("drug consumption" NEAR/2 (room* OR unit* OR facilit* OR galler*))) AND T TOPIC: ((supervise* OR safe OR safer) NEAR/1 (smok* OR inhal* OR snort* OR intranasal* ))

**Cochrane Library** (**Cochrane Database of Systematic Reviews : Issue 9 of 12, September 2017, Cochrane Central Register of Controlled Trials : Issue 8 of 12, August 2017) Searched September 12, 2017 Results=51**

("drug consumption" near/2 (room* or unit* or facilit* or galler*)):ti,ab,kw or ((supervise* or safe or safer) near/1 (smok* or inhal* or snort* or intranasal* or inject*)):ti,ab,kw (Word variations have been searched)

1. Please note that the searches for Medline, EMBASE, and PsycINFO, and CINAHL were updated in August 2020 (limited to the original search date of September 12, 2017) as a minor mistake in the search terms was identified. The numbers provided in the manuscript reflect the corrected search, and the results were not impacted by this correction. [↑](#footnote-ref-1)
