# Supplementary material for: To what extent do supervised drug consumption services incorporate non-injection routes of administration? A systematic scoping review documenting existing facilities
Source: Harm Reduct J. 2020 Oct 7;17:72. doi: 10.1186/s12954-020-00414-y (PMC7539556; doi:10.1186/s12954-020-00414-y)
Supplement: Supplementary file 2 — Additional file 2. A list of all terms used in the grey literature search strategy. [file 12954_2020_414_MOESM2_ESM.docx]

**Additional file 2 Terms used in grey literature search strategy**

1. “supervised injection service”
2. “supervised injection centre”
3. “supervised injection facility”
4. “supervised injection room”
5. “supervised injecting service”
6. “supervised injecting centre”
7. “supervised injecting facility”
8. “supervised injecting room”
9. “supervised inhalation service”
10. “supervised inhalation centre”
11. “supervised inhalation facility”
12. “supervised inhalation room”
13. “supervised inhaling service”
14. “supervised inhaling centre”
15. “supervised inhaling facility”
16. “supervised inhaling room”
17. “supervised consumption service”
18. “supervised consumption centre”
19. “supervised consumption facility”
20. “supervised consumption room”
21. “supervised smoking service”
22. “supervised smoking centre”
23. “supervised smoking facility”
24. “supervised smoking room”
25. “safe injection service”
26. “safe injection centre”
27. “safe injection facility”
28. “safe injection room”
29. “safe injecting service”
30. “safe injecting centre”
31. “safe injecting facility”
32. “safe injecting room”
33. “safe inhalation service”
34. “safe inhalation centre”
35. “safe inhalation facility”
36. “safe inhalation room”
37. “safe inhaling service”
38. “safe inhaling centre”
39. “safe inhaling facility”
40. “safe inhaling room”
41. “safe consumption service”
42. “safe consumption centre”
43. “safe consumption facility”
44. “safe consumption room”
45. “safe smoking service”
46. “safe smoking centre”
47. “safe smoking facility”
48. “safe smoking room”
49. “drug consumption room”
50. “fixing room”
51. “overdose prevention site”
