# Supplementary material for: To what extent do supervised drug consumption services incorporate non-injection routes of administration? A systematic scoping review documenting existing facilities
Source: Harm Reduct J. 2020 Oct 7;17:72. doi: 10.1186/s12954-020-00414-y (PMC7539556; doi:10.1186/s12954-020-00414-y)
Supplement: Supplementary file 3 — Additional file 3. List of exemplar articles used to verify and test the search strategy. [file 12954_2020_414_MOESM3_ESM.docx]

**Additional file 3 Exemplar articles to verify the search strategy**

1. Collins CLC. Potential uptake and correlates of willingness to use a supervised smoking facility for noninjection illicit drug use. J Urban Health Bull N Y Acad Med. 2005 May 11;82(2):276–84.

2. Collins CLC, Kerr T, Tyndall MW, Marsh DC, Kretz PS, Montaner JS, et al. Rationale to evaluate medically supervised safer smoking facilities for non-injection illicit drug users. Can J Public Health. 2005 Sep;96(5):344–7.

3. DeBeck K, Buxton J, Kerr T, Qi J, Montaner J, Wood E. Public crack cocaine smoking and willingness to use a supervised inhalation facility: Implications for street disorder. Subst Abuse Treat Prev Policy. 2011 Feb 23;6(4).

4. Jozaghi E, Vancouver Area Network of Drug Users. A cost-benefit/cost-effectiveness analysis of an unsanctioned supervised smoking facility in the downtown eastside of Vancouver, Canada. Harm Reduct J. 2014 Nov 13;11(30).

5. Kimber J, Dolan K, Wodak A. Survey of drug consumption rooms: Service delivery and perceived public health and amenity impact. Drug Alcohol Rev. 2005 Jan 1;24(1):21–4.

6. McNeil R, Kerr T, Lampkin H, Small W. “We need somewhere to smoke crack”: An ethnographic study of an unsanctioned safer smoking room in Vancouver, Canada. Int J Drug Policy. 2015 Jul;26:645–52.

7. Shannon K, Ishida T, Morgan R, Bear A, Oleson M, Kerr T, et al. Potential community and public health impacts of medically supervised safer smoking facilities for crack cocaine users. Harm Reduct J. 2006 Jan 10;3(1).

8. Watson TM, Strike C, Kolla G, Penn R, Jairam J, Hopkins S, et al. Design considerations for supervised consumption facilities (SCFs): Preferences for facilities where people can inject and smoke drugs. Int J Drug Policy. 2013 Mar;24(2):156–63.
