# Supplementary material for: To what extent do supervised drug consumption services incorporate non-injection routes of administration? A systematic scoping review documenting existing facilities
Source: Harm Reduct J. 2020 Oct 7;17:72. doi: 10.1186/s12954-020-00414-y (PMC7539556; doi:10.1186/s12954-020-00414-y)
Supplement: Supplementary file 4 — Additional file 4. Full details of the screening process including grey literature screening, external reference list screening, and screening for reference list of included articles. [file 12954_2020_414_MOESM4_ESM.docx]

**Additional file 4 Additional details of the screening process**

**Table 1 Grey literature screening**

| **Database** | **Dates Searched** | **Number of Results** | **Number of Results Screened** | **Number of Results Included** |
| --- | --- | --- | --- | --- |
| Google Scholar | October 4 – 26, 2017 | 2,991 | 1,110 | 52 |
| Health Systems Evidence | October 30, 2017 | 1 | 1 | 0 |
| Leading Practices Database | October 30, 2017 | 0 | 0 | 0 |
| Grey Literature Report | October 31, 2017 | 1 | 1 | 0 |
| Archive-It | November 22 – 23, 2017 | 45 | 45 | 6 |
| International Network of Drug Consumption Rooms | November 24, 2017 | 18 | 18 | 5 |
| Harm Reduction International | November 24, 2017 | 16 | 16 | 0 |
| AMICUS | November 24, 2017 | 14 | 14 | 0 |
| European Monitoring Centre for Drugs and Drug Addiction | November 29, 2017 | 3 | 3 | 2 |
| British Library | December 6, 2017 | 108 | 108 | 0 |
| WorldCat | December 6 – 12, 2017 | 716 | 473 | 3 |
| Bielefeld Academic Search Engine | December 12 – 13, 2017 | 858 | 622 | 6 |
| Google | December 14 – 22, 2017 | 162,868 | 2,388 | 36 |
|  |  |  | **Total** | **110** |

**Table 2 External reference list screening**

| **Database** | **Number of Results Screened** | **Number of Results Included** |
| --- | --- | --- |
| Drug War Facts: Supervised Consumption Facilities and Safe Injection Facilities | 40 | 1 |
| Drug Policy Modelling Program: Supervised Injection Facilities – Annotated Bibliography | 134 | 2 |
| Institute of Health Economics: SCS Provincial Evaluation Reference List | 258 | 2 |
|  | **Total** | **5** |

**Table 3 Screening for reference list of included articles**

| **Number of References Screened** | **Number of References Included** |
| --- | --- |
| 14,886 | 22 |
| **Total** | **22** |
